# Childhood asthma is associated with development of type 1 diabetes and inflammatory bowel diseases: a Danish nationwide registry study

**DOI:** 10.1038/s41598-022-26067-4

**Published:** 2022-12-16

**Authors:** Mie Sylow Liljendahl, Astrid Sevelsted, Bo L. Chawes, Jakob Stokholm, Klaus Bønnelykke, Zorana Jovanovic Andersen, Hans Bisgaard

**Affiliations:** 1 COPSAC, Copenhagen Prospective Studies on Asthma in Childhood, Herlev and Gentofte Hospital, University of Copenhagen, Copenhagen, Denmark; 2 Section of Epidemiology, Institute of Public Health, University of Copenhagen, Copenhagen, Denmark

**Keywords:** Asthma, Type 1 diabetes, Inflammatory bowel disease, Epidemiology

## Abstract

Asthma and autoimmune disorders might be affected by opposing immune mechanisms, T helper cells type 2 (Th2) and T helper cells type 1 (Th1) immunity, respectively. Knowledge on comorbidity can increase understanding of the underlying etiologies. We aim to examine the association between childhood asthma and subsequent risk of type 1 diabetes (T1D) and inflammatory bowel diseases (IBD) in Danish children. Children of Danish origin born during 1991–1996 were included and childhood asthma, defined as a minimum of two collected prescriptions of inhalation corticosteroids age 5–7 years, was linked to hospitalisations with either T1D or IBD after age 8. Associations between childhood asthma and incidence of T1D and IBD were analysed using sex- and year stratified Cox regression. A total of 366,200 children were included in the study, 4.9% had asthma, which increased the risk of both T1D and IBD, hazard ratios of 1.32 (1.08–1.61) and 1.27 (1.09–1.48). In this large nationwide Danish study, we found that children with asthma have increased risk of developing immune diseases T1D and IBD. This contradicts the Th1 vs Th2 paradigm and points towards shared disease mechanisms and risk factors.

## Introduction

Asthma is among the leading chronic inflammatory diseases in children globally^1,2^ with early debut^3^ and high prevalence^4^. However, understanding possible relationships between asthma and other less common chronic inflammatory requires large cohorts with long-term follow-up. Such knowledge on comorbidity is important in order to increase understanding of the underlying etiologies.

Type 1 diabetes and inflammatory bowel diseases (Crohn’s disease and ulcerative colitis) are chronic inflammatory diseases, both characterised by increasing prevalence in Westernised countries^2,4,5^, which is also the case for childhood asthma, suggesting potential shared mechanisms. On the other hand, the pathogenesis of type 1 diabetes and inflammatory bowel diseases has been linked to T-cell-mediated responses of primarily T helper cell type 1 (Th1)^4,6^. While a predominance of T helper cells type 2 (Th2) is typically found in asthma and allergic diseases^7^. Based on the supposed counteracting and cross-regulatory properties of Th1 and Th2, it may be assumed that these different diseases will occur in different individuals and that the occurrence of one of the disorders would reduce the risk of developing the other.

Here, we aim to examine whether we see a positive or negative or no association between childhood asthma and the two groups of chronic inflammatory diseases: type 1 diabetes and inflammatory bowel diseases. We used a nationwide population-based cohort of all Danish children and adolescents with birth years 1991–96.

## Methods

### Study population

In this prospective registry-based cohort study, we identified a cohort of all liveborn children of Danish descent born in Denmark in the period 1991–1996 from the Danish Medical Birth Registry^8^. From the birth registry we used information on maternal age, the sex of the child, date of birth, birthweight, parity, and maternal personal identification number^8^. We used the child's unique personal identification number (assigned by the Danish Civil Registration System to all people with permanent residency in Denmark) to link data on collected asthma medication from the Danish National Prescription Registry^9^ and data on hospitalisation outcomes from the Danish National Patient Registry^10^ together with data on date of death, or date of migration away from Denmark from the Danish Civil Registration System^11^. We used the mother’s unique personal identification number to link information on maternal employment status in the year previous to childbirth from Statistics Denmark as a surrogate marker for the child’s socioeconomic status (the highest registered maternal status in 2 years surrounding childbirth categorised as: working; not working; temporarily not working; other). For children who died or emigrated during the study period, this date is noted as the exit date. Children who emigrated (Danish address termination) but returned to Denmark less than 6 months after the emigration, remain included in the study population. Children were observed from age 5 until exit date or final follow-up December 31, 2015 (age 20–25).

### Ethics

The study is based solely on registry data, and no subjects were contacted as part of this study. The study was approved by The Danish Data Protection Agency (j.nr 2012-41-0388). According to Danish legislation no ethics approval or informed consent are needed in register-based studies without direct contact to the participants. There was no patient and public involvement in this study. The project was approved by Statistics Denmark and the national board of health data. All data are accessed via a secure server, data are pseudo anonymised (personal ID numbers are anonymized). The study was conducted according to the principles expressed in the Declaration of Helsinki.

### Childhood asthma

Childhood asthma was defined as a minimum of 2 collections of doctor-prescribed anti-asthmatic inhaled corticosteroids (ICS, ATC codes R03AK06-13, R03BA01-2, R03BA05, and R03BA07) from a Danish pharmacy in the age-range 5 through 7 years. Children with only one ICS collection were included as controls. As sensitivity analysis we examined the severity of asthma by the number of collected ICS prescriptions in the same age interval, testing the linear trend per prescription only among children with at least one collected ICS prescription. Asthma persistence was defined from collected ICS after age 8: a minimum of 2 collections of doctor-prescribed anti-asthmatic inhaled corticosteroids in the age-range 5 through 7 years and minimum two collections after age 8 was labelled as “persistent asthma”, whereas maximum one collection after age 8 was labelled “childhood asthma”. Allergic was defined as a minimum of 2 collections of ICS and a minimum of 2 collections of doctor-prescribed allergy medication, i.e. nasal corticosteroids, nasal, ocular and/or oral antihistamines (ATC codes R01AD** and R01AC**, excluding Cromoglicic acid R01AC01, Cromoglicic acid, combinations R01AC51, and flunisolide R01AD04) from a Danish pharmacy in the same age-range of 5 through 7 years.

### Type 1 diabetes and inflammatory bowel diseases

Cases of type 1 diabetes or inflammatory bowel diseases were defined by first hospitalisation after age 8 with a primary diagnosis (in or out-patient) of either type 1 diabetes (ICD-8 codes before 1994: 249, ICD-10 codes: E10) or inflammatory bowel diseases (ICD-8 codes before 1994: 563 and 56,904, ICD-10 codes: K50-K51). In Denmark ICD-8 codes were used until 1993 subsequently ICD-10 was used from 1994. As sensitivity analyses, we restricted the case definition to only in-patient admissions. For each outcome, children with any record of these diseases prior to age 8 years were excluded from the analysis.

### Reverse association

To investigate the reverse association of subsequent asthma after type 1 diabetes or inflammatory bowel diseases, we defined the study population as all children with no ICS collection in age 5–10. Exposed children of either type 1 diabetes or inflammatory bowel diseases were defined from any hospitalisation with a primary diagnosis (in or out-patient) of either of the respective diagnoses before age 10. Subsequent asthma was defined as a minimum of 2 collections of ICS in a period of 3 years after age 10.

All children with a collection of ICS in the age-range of 5 through 9 years were excluded.

### Covariates

Covariates were chosen a priori as sex, year of birth (one-year groups 1991–1996), cesarean section, parity (first child; second child; third child; fourth child; or more), maternal age (< 20; 20–24; 25–29; 30–34; $\ge$ 35 years), birth weight (< 2.5 kg; 2.5–2.9 kg; 3.0–3.4 kg; 3.5–3.9 kg; 4.0–4.4; 4.5 kg or more), gestational age (< 35 weeks; 35–37 weeks; 38–39 weeks; 40–41 weeks; $\ge$ 42 weeks) and socioeconomic status (the highest registered maternal status in 2 years surrounding childbirth categorised as: working; not working; temporarily not working; other). Furthermore, familiar occurrence of T1D and IBD (first hospitalisation with a primary diagnosis or asthma (a minimum of 2 collections of anti-asthmatic inhaled corticosteroids). Covariates selected for the final adjusted models were based of a variance analysis with a 5% significance level.

### Statistics

Associations between childhood asthma and later development of type 1 diabetes or inflammatory bowel diseases were examined with Kaplan Meier survival functions and Cox regression models. All models were investigated for confounding and proportional hazard assumptions were tested with Schoenfeld residuals. Variables violating the proportional hazard assumption were included in the model as stratifying variables. Thereby the main analysis is a sex-stratified Cox model adjusted for birth year. A stratified Cox model produce one estimate for a predictor, here childhood asthma, while allowing free baseline hazard in different strata, here sex. In a second model we additionally adjusted for socioeconomic status. As sensitivity analysis we restricted the study population to birth years 1992–1996 due to the prescriptions registry only is considered complete for the pediatric population from 1997.

Population attributable risk fraction (PARF) was calculated to assess the overall impact of the exposure on the outcome in the population as p*(HR-1)/(1 + p*(HR-1)), where p is the prevalence of asthma and HR denotes the risk ratio for disease by asthma.

A significance level of 0.05 was used in all analyses. All data processing and analyses were done with R statistical software.

## Results

### Baseline characteristics

406,125 children were born in Denmark during 1991 through 1996. Excluding 30,571 (7.5%) children with non-Danish descent and 9354 children lost to follow-up (migration, death) before the age of 8 years a total of 366,200 children were included in the study population.

Baseline characteristics are presented in Table 1. Cohort year 1994 constituted the largest proportion of the study population (17.2%), and 1991 constituted the smallest proportion (16.0%). The majority of the mothers were 25–29 years (42.3%) and had none (46.6%) or one (36.1%) previous pregnancy. Information on socioeconomic status showed that 85.7% of the mothers were in employment in 2 years surrounding childbirth, while 8.5% were temporarily not working. The remaining 5.0% of mothers were not in employment and finally 0.7% were in the category of others and children (Table 1).

**Table 1:** Baseline characteristics.

	n (%)
Overall	366,200 (100)
**Childhood asthma**	
Yes	18,012 (4.9)
Any diabetes < 8 years	455 (0.1)
Any IBD < 8 years	92 (0.025)
**Birth year**	
1991	58,512 (16.0)
1992	61,382 (16.8)
1993	60,749 (16.6)
1994	62,836 (17.2)
1995	62,690 (17.1)
1996	60,031 (16.4)
**Sex**	
Boy	188,137 (51.4)
Girl	178,063 (48.6)
**Maternal age (year)**	
< 20	12,840 (3.5)
20–24	78,970 (21.6)
25–29	154,452 (42.3)
30–34	90,637 (24.8)
≥ 35	28,570 (7.8)
Missings	731
**Parity**	
0	170,742 (46.6)
1	132,206 (36.1)
2	48,545 (13.3)
3	11,390 (3.1)
4 +	3317 (0.9)
**Gestational (week)**	
< 35	8251 (2.3)
35–37	27,976 (7.7)
38–39	114,931 (31.8)
40–41	179,268 (49.6)
42 +	31,352 (8.7)
Missings	4422
**Birth weight (g)**	
≤ 2499	19,708 (5.4)
2500–2999	42,224 (11.5)
3000–3499	115,479 (31.5)
3500–3999	122,448 (33.4)
4000–4499	54,023 (14.8)
4500 +	12,318 (3.4)
**Socioeconomic status**	
Other and children	2655 (0.7)
Working	313,876 (85.7)
Not working	18,491 (5.0)
Temporarily not working	31,162 (8.5)

Descriptive characteristics of the study population.

Childhood asthma defined by minimum two collected prescriptions of anti-asthmatic ICS in the age-range 5–7 was present in 18,012 (4.9%) of the cohort. The prevalence was similar across the birth years ranging from 4.5% in birth year 1991 to 5.0% in birth year 1996. Of the children with asthma, 69.1% had persistent asthma during follow-up with minimum 2 collected ICS prescriptions after age 8 years. Ten percent of the asthma cases had allergic asthma (N = 2934).

### Type 1 diabetes

A total of 1556 children (0.4%) from the study population had a hospitalisation with type 1 diabetes during follow-up. We excluded 455 children with a debut of type 1 diabetes before 8 years of age from the analysis.

**Figure 1:**
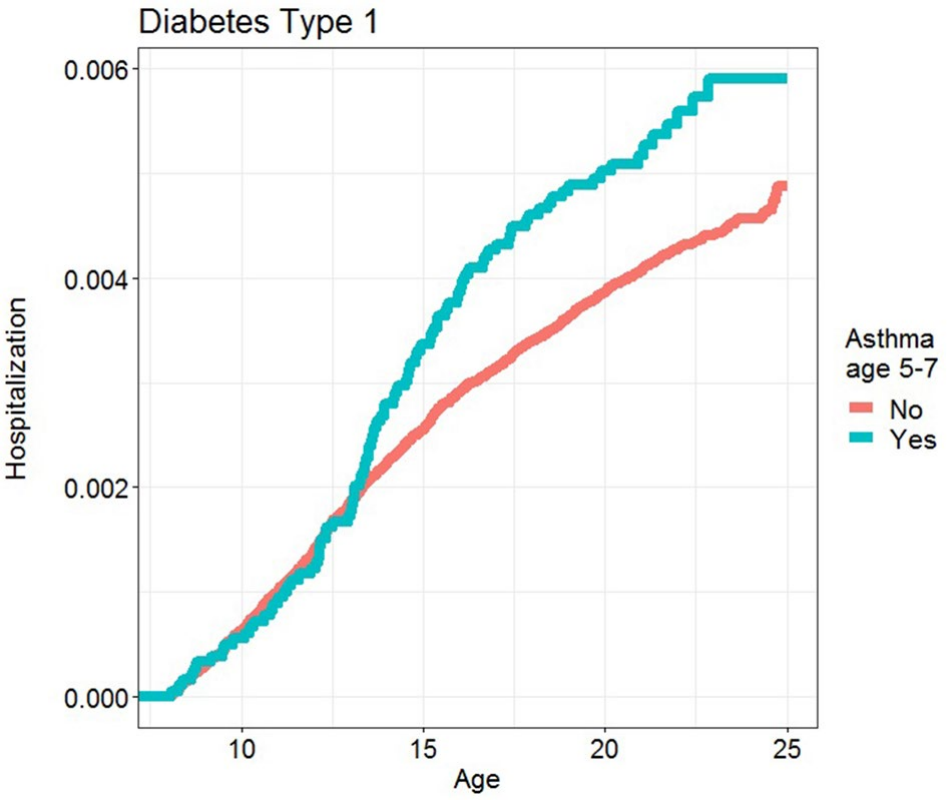
Kaplan Meier curve showing association between childhood asthma and development of type 1 diabetes (p = 0.02).

Childhood asthma was associated with an increased risk of type 1 diabetes: HR 1.28 [95% CI 1.05–1.57; P = 0.021], (Table 2). Kaplan Meier curves are shown in Fig. 1. Adjustment for all covariates did not substantially change the estimate: adjusted HR 1.23 [1.00–1.52; P = 0.055] (Supplementary Table E1). The strongest effect was seen among boys HR 1.35 [1.05–1.72; P = 0.017] compared to girls 1.10 [0.76–1.61; P = 0.061], however there were no significant interaction between sex and asthma, P = 0.38, Table 3.

**Table 2:** Hazard ratios by childhood asthma for T1D and IBD.

Outcome	n cases/n total	HR [95% CI]
Type 1 diabetes	1556/364,189	1.28 [1.05–1.57]
Inflammatory bowel diseases	2086/366,108	1.26 [1.05–1.51]

Associations between childhood asthma and development of chronic inflammatory diseases: hazard ratios by childhood asthma for T1D and IBD. All models are stratified for sex and adjusted for birth year.

**Table 3:** Hazard ratios by childhood asthma for T1D and IBD.

Outcome	Boys HR [95% CI]	Girls HR [95% CI]	P value for interaction
Type 1 diabetes	1.35 [1.05–1.72]	1.10 [0.76–1.61]	0.38
Inflammatory bowel diseases	1.38 [1.10–1.74]	1.09 [0.80–1.47]	0.21

Sex specific associations between childhood asthma and development of chronic inflammatory diseases: hazard ratios by childhood asthma for T1D and IBD. All models are stratified for sex and adjusted for birth year.

Results were similar when only inpatient hospitalisations for asthma were used for case definition, but not significant (Supplementary Table E2). We found no dose–response effect of the severity of asthma, defined by the number of collected ICS prescriptions at age 5–7 (Supplementary Table E3). However, we found stronger associations with type 1 diabetes among asthmatics with persistent asthma, defined as continued collections of ICS after age 8: HR 1.30 [1.02–1.65; P = 0.034] vs childhood asthma: HR 1.19 [0.82–1.73]; P = 0.358 (Supplementary Table E4), although not statistically significant (P = 0.75). We found no increased risk of type 1 diabetes among the smaller subset of children with allergic asthma (Supplementary Table E5).

Population attributable risk fraction showed that 1.54% of type 1 diabetes cases can be explained by childhood asthma.

### Inflammatory bowel diseases

A total of 2064 (0.6%) children from the study population had at least one hospitalisation with inflammatory bowel diseases during follow-up, equally distributed between Crohn's disease and ulcerative colitis. Only 92 children with a debut of inflammatory bowel diseases before 8 years of age and were excluded from the analyses.

**Figure 2:**
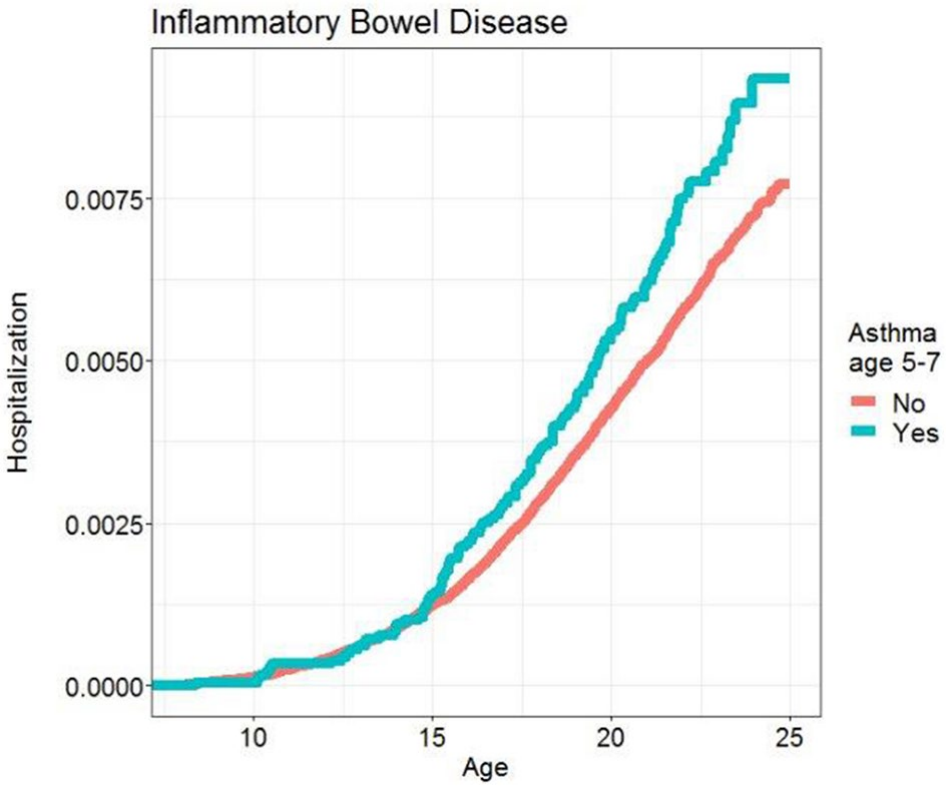
Kaplan Meier curve showing association between childhood asthma and development of inflammatory bowel disease (p = 0.01).

Childhood asthma increased the risk of inflammatory bowel diseases: HR 1.26 [1.05–1.51; P = 0.012] (Table 2 and Fig. 2) and in the model including all the covariates 1.28 [1.06–1.53; P = 0.008] (Supplementary Table E1). Again the effect of childhood asthma was mainly found among boys, HR 1.38 [1.10–1.74; P = 0.005] compared to girls 1.09 [0.80–1.47; P = 0.592], though no interaction were found between childhood asthma and sex (p = 0.21) (Table 3). We found similar risk in both Cronh’s disease with HR 1.24 [0.97–1.58; P = 0.094]; and ulcerative colitis 1.30 [1.03–1.64; P = 0.027].

The majority of the cases were out-patients, and when restricting case definition to only inpatient hospitalisations (N reduced from 2086 to 789) we found no association with childhood asthma (Supplementary Table E2). Opposite to type 1 diabetes there was a tendency towards a dose–response effect of asthma severity in the age-range of 5–7 years; P = 0.21 (Supplementary Table E3). Similar to type 1 diabetes the association was primarily driven by asthmatics with persistent asthma: HR 1.45 [1.18–1.77; P < 0.001] vs no association from childhood asthma: HR 0.86 [0.58–1.26; P = 0.427] (Supplementary Table E4), the difference was significant (P = 0.02). Allergic asthma did not show an increased risk: HR 1.14 [0.63–2.07] (Supplementary Table E5).

The impact on population level calculated by the population attributable risk fraction showed that 1.25% of inflammatory bowel disease cases can be explained by childhood asthma.

### Sensitivity analyses

Supplementary Table E6 shows the results excluding birth year 1991 from the study population, since the prescriptions registry only is considered complete for the pediatric population from 1997. Overall, the associations remained significant but with fewer cases and smaller effect estimates.

### Reverse association

In an analysis of the reverse association, i.e. if either type 1 diabetes or IBD alters the risk of subsequent asthma we included 331,450 children who were free of asthma 5 through 9. Among these children 607 (0.2%) had any diabetes and 118 (0.04%) had any IBD hospitalisation before age 10. In total 15,379 (4.9%) were defined as asthmatics after age 10 with at least two collections of ICS in a period of three years. Any type 1 diabetes significantly reduces the risk of later asthma: HR 0.52 [95% CI 0.31–0.68], and associations were similar although non-significant for IBD: HR 0.73 [95% CI 0.27–1.95], Table 4.

**Table 4:** Hazard ratios by T1D and IBD for asthma.

	Asthma cases with immune disease	Asthma cases without immune disease	HR [95% CI]	P-value
Type 1 diabetes	15/607 (2.47%)	15,379/315,499 (4.88%)	0.52 [0.31—0.68]	0.011
Inflammatory bowel diseases	4/118 (3.39%)	15,390/331,332 (4.64%)	0.73 [0.27—1.95]	0.532

Associations between inflammatory diseases at age 10 years and later asthma development: hazard ratios by T1D and IBD for asthma.

## Discussion

In this nationwide Danish registry study, we found childhood asthma to be associated with higher risk of development of two different chronic inflammatory diseases; type 1 diabetes and inflammatory bowel diseases. Childhood asthma increased the risk of both diseases by 20–30%, which was robust for case definitions, confounder adjustments, and stratifications. The associations seemed more pronounced in boys and among children with persistent asthma, though we did not observe significant sex interactions.

These associations are interesting from an aetiological and mechanistic viewpoint, since the diseases have different pathology, clinical presentations and our findings thereby dismiss the null hypothesis that these differing inflammatory trajectories cannot co-occur.

Denmark is uniquely placed to facilitate large nationwide registry studies because the health service is tax-funded, practically all diagnoses are registered in publicly funded hospitals, all diagnoses are centrally recorded, and all patients are identifiable by a unique personal identification number, which makes precise data linkage possible. Especially for the pediatric population there are no private alternatives to the public hospitals.

We excluded children not born in Denmark to ensure a study population with similar treatment options and to not miss any potential diagnosis of type 1 diabetes or inflammatory bowel diseases before migration to Denmark. In addition, we excluded children with non-danish descent to ensure a more genetically homogeneous population, since the diseases under investigation all have strong genetic components^12–14^.

The study population is limited to a few birth years, which is mainly because of the data availability. The Danish National Prescription Registry was initiated in 1994 but is only considered complete for the pediatric population around 1997 where almost all of the pediatric population collects medication on their own personal ID card (as opposed to collecting on a parent ID card). To define childhood asthma from prescription medication and allow follow-up time to investigate the chronic inflammatory diseases we restricted our population by birth year. The first birth year 1991 therefore relies in part on data from the less certain year 1996, and we also found less asthma cases in this birth year suggesting some misclassification, where asthmatic children are potentially defined as controls in this year. Such misclassification would bias the results towards null; however, we did find associations with type 1 diabetes and inflammatory bowel diseases when excluding birth year 1991 indicating that any bias in year 1991 was not towards null. The differences in effect estimates are most likely due to shorter follow-up and thereby lesser cases without year 1991.

It can be difficult to diagnose asthma in young children, e.g. lung function test are not feasible before school-age. The age-range 5–7 years were based on the consideration of a more age-specific asthma definition than previously used in Nordic studies and data availability in the Danish national registries, including follow-up time. Children were observed from age 5 until exit date or final follow-up December 31, 2015 (age 20–25). The unclear definition of childhood asthma and the differences among the existing literature make it difficult to compare results. Despite the limitations in the use of ICS, the Danish national registers provide a well-defined asthma population that covers children with active asthma in an age group where the diagnosis is more certain than in younger children.

The limited follow-up time (latest available data are from 2015) ranging from age 19 to 25 also restricts our case definitions since both type 1 diabetes and inflammatory bowel diseases can debut later than age 25^15^. Thus, some controls will change to cases at a later time point, which is an obvious limitation of our study but would introduce bias towards null. It is not certain whether this bias is non-differential, i.e. if the childhood asthma phenotype is particularly associated with specific debut ages of the chosen diseases. Future studies should investigate this.

The results only represent an ethnically homogeneous population of Danish origin. The results cannot be generalised to other populations of other ethnicity as this group was excluded from the study population.

For type 1 diabetes and inflammatory bowel diseases we excluded cases with debut before age 8. This exclusion is made on the basis that the exposure to childhood asthma was defined by prescriptions in the age-range 5–7 year, and outcomes should be observed subsequent to the exposure. However, we cannot exclude that some of the children defined as controls in the study later on are defined as asthma cases (collections of prescription of anti-asthmatic inhalation corticosteroids in the ages before 5) or simply have mild symptoms that require b2 agonist but not ICS. However, this would work for the null hypothesis. Conversely, some of the controls may have had asthma in the past but no longer need ICS in the age 5–7 years. Unfortunately, we did not have suitable information to study the allergic asthma phenotype more detailed. Allergic asthma might only include a subgroup of TH2 asthma due to data availability. Allergic asthma included allergy medication, i.e. nasal corticosteroids, nasal, ocular and/or oral antihistamines.

Our results are in agreement with previous Finish^2^ and Swedish^16^ prospective nationwide registry based studies showing a positive association between childhood asthma and type 1 diabetes, but also a negative reverse association where type 1 diabetes and the risk of subsequent asthma. Furthermore, a Norwegian study found an increased occurrence of chronic inflammatory diseases including mental illness (attention deficit/hyperactivity disorder, epilepsy and migraine cardiovascular disease, diabetes, autoimmune disorders, gastro-oesophageal reflux disease, and allergy) in the population with asthma. The study was a cross-sectional study of an asthma population aged 8–29 year^17^. Another cross sectional study on inflammatory bowel disease patients found asthma, psoriasis and type 1 diabetes to be the most important comorbidity conditions among the patients^18^, and smaller studies have also reported an increased risk of inflammatory bowel disease among asthma patients^18–20^. Our study differentiates from the other Nordic studies in a more careful approach to the temporal associations and a more age-specific asthma definition.

Together, these studies question the paradigm of counter-regulation of Th1 and Th2 inflammatory pathways, which predicted that Th2 mediated asthma would not co-occur with Th1 mediated diseases such as type 1 diabetes and inflammatory bowel diseases when asthma is the first occurring disease—but not when the Th1 mediated diseases occur first.

Alternative explanations for our findings could be shared genetic risk factors and shared environmental risk factors. Asthma and type 1 diabetes have strong genetic components. Twin studies have estimated 75% of the variation in propensity to asthma was explained by genetic effects^21^, and up to 85% for type 1 diabetes^22^. In contrast inflammatory bowel diseases are far less heritable, and with stronger heritability in Crohn’s disease around 50% than ulcerative colitis around 15%^14,23^. We previously found shared susceptibility loci and commonalities in pathways between allergy and autoimmune diseases, suggesting shared disease mechanisms which could explain the associations^24^. Interestingly, overlapping genetic loci showed examples of gene variants with same direction of effect as well as opposite directions of effect, suggesting potential underlying genetic mechanisms related to both shared disease pathways increasing risk of these disorders as well as opposing/”polarizing” mechanisms potentially leading to inverse comorbidity patterns.

Additionally, to the shared genetic risk factors, shared environmental risk factors may explain the communality. As we previously demonstrated, cesarean section is a common risk factor for both asthma and inflammatory bowel diseases, but interestingly not for type 1 diabetes after adjustment for heritage^15^. Furthermore, parental smoking during pregnancy or in early life of the child is a consistent risk factor for childhood asthma^3,25^ and also inflammatory bowel diseases^26^. In general asthma has been associated with a wealth of pre- and perinatal risk factors pretubating the developing inflammatory system. Some early life risk factors may have lingering effects on the inflammatory system and cause diseases later in childhood, adolescence and early adulthood.

The systemic influence of inhaled steroids is still discussed controversy, and there is discrepancy in the existing literature on the effect of ICS on glucose metabolism in diabetic individuals. Some studies clearly demonstrated an association of dysglycemia and high dose ICS. Other studies failed to demonstrate clinically significant adverse dysglycemic effects of ICS treatment^27–30^. This is pathomechanisticly primarily interesting in patients with type 2 diabetes and the association are not observed in patients with type 1 diabetes; future studies should investigate this.

It is possible that the order of debut of asthma compared to autoimmune disorders might be essential. This is suggested from a Finnish study finding the same positive correlation between asthma and later development of type 1 diabetes, but a lower risk of asthma if type 1 diabetes occurred first^1^, and the same inverse correlation was seen between occurrence of autoimmune antibodies and later asthma and eczema^31^.

Together, these findings suggest complex and shared aetiology of chronic inflammatory diseases despite their different pathology and clinical presentation. It is important to note that although the associations may have importance for the understanding of complex disease aetiology, the estimated population attributable risk fractions for both type 1 diabetes and inflammatory bowel diseases were small, and the clinical relevance for individual patients is negligible. Still, our findings imply that an efficient prevention of childhood asthma; e.g. micronutrient supplementation in pregnancy^32,33^ may also reduce the risk of other chronic inflammatory diseases.

## Conclusion

This nationwide Danish registry-based study showed an increased risk of development of both type 1 diabetes and inflammatory bowel diseases for children with childhood asthma at age 5–7 years. These results largely dismiss the simple Th1 vs Th2 explanations for these diseases and suggest common mechanisms for these three chronic inflammatory diseases.

## Supplementary Information

Supplementary Information.

## Data Availability

The data that support the findings of this study are available from Statistics Denmark, but restrictions apply to the availability of these data, which were used under license for the current study, and therefore data are not publicly available. Data are however available from the authors upon reasonable request and with permission of Statistics Denmark. Registry data are protected under the Danish Data Protection Act and European Regulation 2016/679 of the European Parliament and of the Council (GDPR) that prohibit distribution even in pseudo-anonymized form. Only authorized (by Denmarks Statistics) research institution can be granted access to data. Please contact the corresponding author for a data analysis agreement as a collaboration effort. We are aware of and comply with recognised codes of good research practice, including the Danish Code of Conduct for Research Integrity. Privacy is important to us which is why we follow national and international legislation on General Data Protection Regulation (GDPR), the Danish Act on Processing of Personal Data and the practice of the Danish Data Inspectorate.
