# Supplementary material for: Childhood asthma is associated with development of type 1 diabetes and inflammatory bowel diseases: a Danish nationwide registry study
Source: Sci Rep. 2022 Dec 16;12:21728. doi: 10.1038/s41598-022-26067-4 (PMC9758130; doi:10.1038/s41598-022-26067-4)
Supplement: Supplementary file 1 — Supplementary Information. [file 41598_2022_26067_MOESM1_ESM.pdf]

Childhood Asthma is Associated with Development of Type 1 Diabetes and Inflammatory Bowel Diseases - a Danish Nationwide Registry Study

Mie Sylow Liljendahl, MSc; Astrid Sevelsted, MSc, Ph.D; Bo L. Chawes, MD, PhD, DMSc; Jakob Stockholm, MD, Ph.D; Klaus Bønnelykke, DMSc; Zorana Jovanovic Andersen, Ph.D, Hans Bisgaard, Professor, DMSc, MD.

Supplementary

Table E1: Fully adjusted

Associations between childhood asthma and later inflammatory diseases: Hazard Ratios by childhood asthma for T1D and IBD. The model is stratified for sex and adjusted for birth year, socioeconomic status, maternal age, birth weight, cesarean section, familiar occurrence of T1D/IBD or asthma.

**Outcome**	**N cases / N total**	**HR<br>[95% CI]**
**Type 1 diabetes**	1,550 / 364,195	1.23<br>[1.00 - 1.52]
**Inflammatory bowel diseases**	2,057 / 360,988	1.28<br>[1.06 - 1.53]

Table E2: Inpatient outcomes

Associations between childhood asthma and later inflammatory diseases: Hazard Ratios by childhood asthma for T1D and IBD, defined exclusively from inpatient hospitalisations. All models are stratified for sex and adjusted for birth year

**Outcome**	**N cases / N total**	**HR**<br>**[95% CI]**
**Type 1 diabetes**	1,361 / 364,384	1.20<br>[0.96 - 1.50]
**Inflammatory bowel diseases**	789 / 366,112	1.06<br>[0.77 - 1.46]

Table E3: Severity of asthma

Associations between childhood asthma in different severities and later inflammatory diseases: Hazard Ratios by childhood asthma for T1D and IBD. All models are stratified for sex and adjusted for birth year

Outcome	1 collected prescriptions HR [95% CI]	2 collected prescriptions HR [95% CI]	3 collected prescriptions HR [95% CI]	4 collected prescriptions HR [95% CI]	>4 collected prescriptions HR [95% CI]	P-value for trend (only children with ICS)
**Type 1 diabetes**	1.18 [0.98 - 1.41]	1.27 [1.03 - 1.56]	1.28 [1.02 - 1.61]	1.21 [0.93 - 1.56]	1.19 [0.90 - 1.58]	0.83
**Inflammatory bowel diseases**	1.27 [1.09 - 1.48]	1.26 [1.05 - 1.51]	1.31 [1.07 - 1.60]	1.44 [1.17 - 1.78]	1.40 [1.11 - 1.76]	0.21

Table E4: Asthma persistence

Associations between childhood asthma and later inflammatory diseases stratified by later asthma defined by collected ICS after age 8. Asthma children with minimum 2 collections after age 8 are labelled "persistent", vs asthma children with maximum 1 collection after age 8 are labelled "childhood asthma", both are compared to children with no asthma 5-7: Hazard Ratios by childhood asthma for T1D and IBD. All models are stratified for sex and adjusted for birth year

**Outcome**	**Childhood asthma HR [95% CI]**	**Persistent asthma HR [95% CI]**	**P value between childhood asthma and persistent asthma**
**Type 1 diabetes**	1.19 [0.82 - 1.73]	1.30 [1.02 - 1.65]	0.75
**Inflammatory bowel diseases**	0.86 [0.58 - 1.26]	1.45 [1.18 - 1.77]	0.02

Table E5: Allergic asthma

Associations between childhood allergic asthma and later inflammatory diseases: Hazard Ratios by childhood asthma for T1D and IBD. All models are Stratified for sex and adjusted for birth year

**Outcome**	**N cases / N total**	**HR**<br>**[95% CI]**
**Type 1 diabetes**	1,459 / 361,264	0.71<br>[0.37 - 1.37]
**Inflammatory bowel diseases**	2,064 / 666,112	1.14<br>[0.63 - 2.07]

Table E6: Excluding year 1991

Associations between childhood asthma and later inflammatory diseases: Hazard Ratios by childhood asthma for T1D and IBD. All models are Stratified for sex and adjusted for birth year

**Outcome**	**N cases / N total**	**HR**<br>**[95% CI]**
**Type 1 diabetes**	1,301 / 305,984	1.26<br>[1.01 - 1.58]
**Inflammatory bowel diseases**	1,657 / 307,618	1.27<br>[1.03 - 1.55]
